# First Transcriptome Analysis of Iranian Scorpion, *Mesobuthus Eupeus *Venom Gland

**Published:** 2018

**Authors:** Masoumeh Baradaran, Amir Jalali, Maryam Naderi-Soorki, Mahmoud Jokar, Hamid Galehdari

**Affiliations:** a *Toxicology Research Center, Ahvaz Jundishapur University of Medical Sciences, Ahvaz, Iran.*; b *Department of Toxicology, School of Pharmacy and Toxicology Research Center, Ahvaz Jundishapur University of Medical Sciences, Ahvaz, Iran.*; c *Genetics Department, School of Sciences, Shahid Chamran University of Ahvaz, Ahvaz, Iran. *; d *Cotton Research Institute of Iran, Agricultural Research, Education and Extension Organization (AREEO), Gorgan, Iran.*

**Keywords:** cDNA library, *Mesobuthus eupeus*, Transcriptome analysis, Chlorotoxin-like peptide, Homology modeling, Molecular dynamics flexible fitting (MDFF)

## Abstract

Scorpions are generally an important source of bioactive components, including toxins and other small peptides as attractive molecules for new drug development. *Mesobuthus eupeus*, from medically important and widely distributed *Buthidae* family, is the most abundant species in Iran. Researchers are interesting on the gland of this scorpion due to the complexity of its venom. Here, we have analyzed the transcriptome based on expressed sequence tag (EST) database from the venom tissue of Iranian *M. eupeus* by constructing a cDNA library and subsequent Sanger sequencing of obtained inserts. Sixty-three unique transcripts were identified, which were grouped in different categories, including Toxins (44 transcripts), Cell Proteins (9 transcripts), Antimicrobial Peptides (4 transcripts) and Unknown Peptides (3 transcripts). The analysis of the ESTs revealed several new components categorized among various toxin families with effect on ion channels. Sequence analysis of a new precursor provides evidence to validate the first CaTxs from *M. eupeus*. The results are exploration of the diversity of precursors expressed of Iranian *M. eupeus* venom gland. We further described comparative analysis of venom components of Iranian *M. eupeus* with other sibling species.

## Introduction

Iranian scorpion’s fauna has two families, *Buthidae *and *Scorpionidae*. Of those, at least 7 species (*Hemiscorpius *lepturus, Odontobuthus doriae, Buthotus salcyi, Buthotus sach, Andrectonus crassicauda, Apisthobuthus petrigus and Mesobuthus eupeus) are implicated in envenoming of humans. *Mesobuthus eupeus* is widespread in Iran and also in neighbor’s countries. It was demonstrated that 45% of scorpion stings in Iran were due to *Mesobuthus eupeus*. The venom of this scorpion contains several compounds, which may cause a number of sting symptoms (1-3). 

Scorpion’s venom is a mixture of different peptides, enzymes, amines, lipids, and other yet unknown bioactive compounds (4). Toxins and antimicrobial peptides are two most medically important groups of venom peptide. Toxins mostly block or modify ion channels (Na^+^, Ca^+2^, K^+^, Cl^-^) (5), while antimicrobial peptides can impair development of microbes. Antimicrobial peptides exhibit a broad-spectrum of activity against Gram-positive bacteria, Gram-negative bacteria and fungi by causing membrane lyses (6). Due to the large variety of structure and function, venom peptides are suggested as new drugs for treatment of various diseases including inflammatory, hematological, autoimmune, infections, cardiovascular, neuromuscular, and psychotic disorders. Accordingly, venom components can be used as pharmaceutical tools (e.g., to characterize ion channels), or biotechnological applications (e.g., insecticides) (4). 

To date, more than 300000 different peptides are considered in the scorpion venoms, and until now still 1% of total peptides are identified (7). Therefore, there is a lot of information in the scorpion venom that is waiting to be discovered.

Bioinformatics-aided Venomics, known as venom informatics, is the complementary part of experimental studies being helpful in identification and classification of the new venom peptides and reduction of the further experimental processes (8).

Iranian *M. eupeus* venom is partly characterized using fractionation, chromatography, mass spectrometry, and peptide sequencing (3, 9), but analysis of transcriptome or Expressed Sequence Tags (ESTs) still remains undiscovered. 

We presented a comprehensive study of transcriptome analysis of Iranian* M. eupeus* venom gland by cDNA library construction leading to identification of the main and new putative venom peptides expressed in the venom glands, some of which may be potent targets of new therapeutic agents.

## Experimental

*Sample preparation and RNA extraction*

Scorpions *Mesobuthus eupeus *were collected from the southwestern province of Iran, Khuzestan and identified by Toxicology Research Center laboratory of Ahvaz Jundishapur University of Medical Sciences. The scorpions were milked three days prior to RNA extraction. The milking was carried out to allow the toxin - producing cells of the venom glands to enter the secretary phase**.**

Total RNA was extracted under sterile conditions from 20 cut homogenized venom glands according to protocol instruction of RNeasy Mini Kit (Cat. 74104) and QIA shredder (Cat. 79654). Nanodrop (co. Thermo, USA) was used for measure RNA concentration.

*cDNA library construction*

SMART (Switching Mechanism At 5’ end of RNA Template) cDNA synthesis technology is a PCR-based method for cDNA library construction that is designed to preferentially enrich for full-length cDNAs. CDNA synthesis was carried out using In-Fusion® SMARTer® Directional cDNA Library Construction Kit (Cat. No. 634933) from 123ng extracted RNA. The In-Fusion SMARTer Directional cDNA Library Construction Kit provides a dependable method for producing high-quality full-length cDNA libraries.

*Cloning of synthesized cDNA in to the pSMART2IFD vector*

The synthesized cDNA was cloned into the pSMART2IFD Linearized Vector, according to kit instrument. The location of the cloning site would be within lacZα. So, this allows for blue/white selection (i.e., α-complementation) after cloning. 

*Transformation into chemical competent E.coli*

*Ecoli *cells (DH5α strain) were chemically competent with CaCl_2_ and heat shock for transformation of the vector including the cDNA insert, to achieve high efficiency output. Transformed cells were grown on LB agar plate containing 100 µg/mL Ampicillin, 1 mM IPTG, and 75µg/mL X-Gal.

*Screening the cDNA library*

Screening of the cDNA library was done in three steps. In step one; white colonies were selected because they have inserts (blue/white selection). In step two, colony PCR was done for each white colony by Forward Screening Primer (TCACACAGGAAACAGCTATGA) and Reverse Screening Primer (CCTCTTCGCTATTACGCCAGC) complemented to pSMART2IFD Linearized Vector in the blunt ends flanking the insert site. With this strategy presence of insert would be checked. Plasmids with DNA insert more than 700 bp in this PCR are carrying a cDNA insert corresponding to putative venom peptide transcripts. These plasmids subsequently in step tree were extracted using QIAprep Spin Miniprep Kit (Cat. 27104) and their sequences were determined by Sanger method from both ends using specific Forward (TCACACAGGAAACAGCTATGA)and Reverse(CCTCTTCGCTATTACGCCAGC) primers by ®Microgen laboratory sequencing service (south Korea). 

*Bioinformatics analysis of transcriptome*

Plasmids containing ESTs without significant similarity to any other ESTs were labled as sigles. subsequently the nucleotide sequences corresponding to vector, adaptors, and Escherichia coli DNA, and any short transcripts (< 100bp)were removed by vecscreen tool (https://www.ncbi.nlm.nih.gov/tools/vecscreen/) and the SeqClean program(http://compbio.dfci.harvard.edu/tgi/software).The final sequences were deposited in GenBank under certain accession numbers. Amino acid sequence of the transcripts was deduced using the Open Reading Frame (ORF) finder program (http://www.ncbi.nlm.nih.gov/projects/gorf/). Sequence of ORFs was confirmed with protein BLAST program on NCBI (http://www.ncbi.nlm.nih.gov/) and UniProt (http://www.uniprot.org/) servers. UniProt server was also used for protein sequence alignments. Signal peptides are predicted for putative venom components by signalP4.1 available on http://www.cbs.dtu.dk/services/SignalP/. Disulphide bond patterns were determined by DISULFIND online server (10). Finally, some unknown data were analyzed by Phyre2 server (11) for domain analysis, structural, and functional prediction.

## Results and Discussion

A raw cDNA library was constructed from Iranian *M. eupeus* venom glands. More than 350 clones were observed after transformation. Most of the clones were white and positive, as high efficiency of library construction. 250 clones randomly were screened to check the insert sequences. The sequences of plasmids of all 250 samples were determined using Sanger sequencing method. After removing short and low quality sequences, we obtained 198 high-quality ESTs with the length between 200bp to 800bp as putative sequences of Iranian *M. eupeus *venom components. All obtained sequences were analyzed by ORF finder and BLAST to remove iterative sequences. Some of cDNA sequences (19 sequences) have incomplete ORF (no start or stop codons). These sequences were named as “no ORF”. Finally, we identified 63 unique sequences from remaining 179 sequences. Among those, 4 sequences have no similarity with currently known sequences available in database. They are grouped as “No match”(6%). Putative ORF (the deduced amino acid sequence) analysis of remaining 59 sequences revealed some similarity with known venom components relative to “Toxins”(KTxs, NaTxs, ClTxs and other toxins, comprising 44 sequences corresponding to 69%), “Cell proteins”(comprising 9 sequences corresponding to 14%), and “Antimicrobial Peptides” (comprising 4 sequences corresponding to 6%). Two of ORF sequences are similar to hypothetical proteins. In fact, they are novel peptides that need further analysis and functional assay. They are categorized as “Unknown Peptides” (5%). All identified cDNA sequences were deposited in GenBank under certain ID, as are given in the Additional file: Table S1. Some of identified ESTs have some similarity with peptides previously reported in Russian or Chinese *M. eupeus*. Therefore, it suggests that the Iranian *M. eupeus* belongs to different subspecies from Russian or Chinese *M. eupeus* because of species diversity and geographical distance. The ESTs labeled as “new”in the Additional file, Table S1, are reported for the first time from *M. eupeus* species.

From all of here analyzed sequences, 12 sequences (meuCLAP, meuVNP2, meuVNP3, meuVAP-6, meuAP-18-1, meuTx19, meuVNP-1A, meuTx20, meuTx23, meuVAP-1, meuVNP-1B and meuTx33) have any Cysteine residues. The scorpion venom peptides based on the having Cysteine residue and ability to building disulphide bridge are generally classified into two main groups: the disulfide-bridged peptides (DBPs), which have three to four disulfide bridges formed each by two cysteine residues; and a smaller group of scorpion venom components, the non-disulfide-bridged peptides (NDBPs), that have any cysteine residues and display multifunctional activities (12). Therefore, the above-noted 12 sequences identified in Iranian *M. eupeus* venom gland transcriptome were classified in NDBPs. The other identified peptides are comprised 2 to 13 cysteine residues that the patterns of their disulphide bonds are shown in Additional file: Table S1 (last column). Proportionally, NDBPs compose a relatively small part of *M. eupeus* venom peptides (20%). It is consistent with the previous study that reported NDBPs as a small group of scorpion venom arsenal (13)

**Figure.1:**
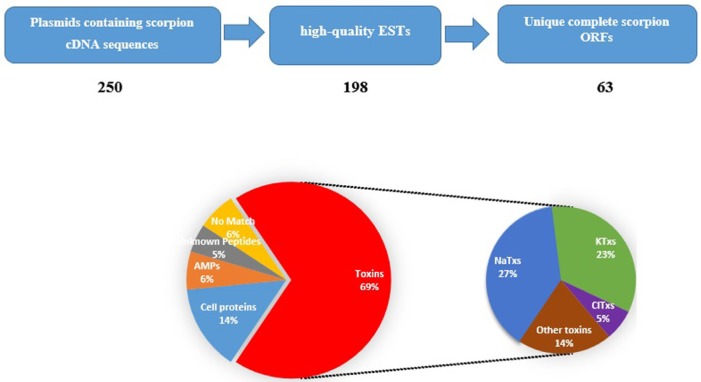
**Stages of identification of putative components in the Iranian **M**. **eupeus **cDNA library, and** relative proportion of the different transcript categories. Raw cDNA sequences analyzed for removing short and low-quality sequences. Remaining high-quality ESTs were analyzed for finding unique sequences. Then scorpion sequences were divided in five groups. Eventually, Toxin group separated in to four subgroups

Figure 1 shows proportions of different groups of identified venom components of Iranian *M. eupeus*. Each group venom components are separately described below.

*Toxins*

Toxins are toxic peptides that produce in venoms and secreted in poisons. Toxins have specific actions on biological systems (14). The most frequent transcripts in the present study is related to toxins (70%). It is consistent with the poisonous nature of the scorpion venom gland. As shown in Figure 1 a total of 44 toxin sequences were identified, from which sodium channel toxins (NaTxs) (27%) are the most frequent. KTxs (23%) and ClTxs (5%) are in second and third places, respectively. 9 transcripts of toxins (14%) are related to “other toxins” (Figure 1). 

*M. eupeus Sodium Channel Toxins*

Voltage-gated sodium channels (VGSCs) are large integral membrane proteins, which are critical for the initiation and propagation of action potential in excitable cells (15). Thereby scorpion Sodium channel toxins (NaTxs) that modify the activity of VGSCs are the most medically important type of scorpion toxins. These toxins are 6500–8500 Da containing 58–76 amino acid residues and linked by 4 disulfide bridges through 8 cysteine residues (7). 

Here, 17 ESTs were identified eventually relating to sodium channel modifier. The mature peptide of detected NaTxs is 52-87 amino acid residues in length. Physiologically, NaTxs are divided into two groups: alpha-toxins (α-NaTxs) and beta-toxins (β-NaTxs). α-NaTxs bind to receptor site 3 of the VGSCs and inhibit the inactivation of the channel (16), whereas β-NaTxs bind to receptor site 4 and shift the threshold potential needed to activate the channel to more negative voltages. According to their different pharmacological preferences, α-NaTxs can be divided into three subgroups: classical α, α-like, and insect α-toxins. Classical α-toxins are very toxic to mammals. Insect α-toxins are especially toxic for insects. The α-like toxins act on both mammals and insects (17). In consonance with in-vivo effects, β-NaTxs selective on insects are classified as “excitatory” or “depressant”, and β-NaTxs which target mammalians VGSCs are classified as β-mammals (18).

Eight of identified NaTxs in this cDNA library, including meuTx17 (ID: KU569303), meuTx18 (ID: KU569301), meuNaTx-13(ID: KU316189), meuNaTx-4 (ID: KU316195), meuNaTx-5(ID: KU316196), meuNaTx-1(ID: KU316191), meuNa32 (ID: KU513855), meuNaTx-2(ID: KU513840) are ɑ-type. Other 9 NaTxs are β-type from which meuNa6 (ID: KU316186), meuNa7 (ID: KU316187), meuNa10 (ID: KU316194), meuNa11 (ID: KU316193), meuNa12 (ID: KU316192) and meuTx21 (ID: KU513851) match depressant toxins, while meuNa8 (ID: KU316188), meuNa9 (ID: KU316190), meuNa13 (ID: KU316197) are matched to excitatory toxins.

From all detected NaTxs, meuNa6, meuNa7, meuNa8, meuNa9, meuNa10, meuNa11, meuNa12, meuNa13, meuTx17, meuTx18, and meuTx21 are firstly reported in *M. eupeus* (Additional file: Table S1).

Except for the meuNa8, meuNa9, meuNa13, meuNaTx-1, and meuTx18, all other identified NaTxs consist of a putative conserved domain belonging to the Toxin-3 superfamily. This family contains both neurotoxins and plant defensins. The neurotoxins of this family bind to sodium channels and inhibit the activation mechanisms of the channels, thereby blocking neuronal transmission (19).

*M. eupeus Potassium Channel Toxins*

Voltage-gated potassium channels (VGPCs) with activation during action potentials in neurons play a major role in action potential, repolarization, and repetitive firing (20); therefore, modulation of this ion channels can effect on function of neurons and cause the disease. Scorpion toxins targeting K^+^-channels (KTxs) control electrical excitability as well as the resting membrane potential in many different cell types. They have been used as tools for characterization of potassium channels and represent important pharmacological tools and potential drug candidates (21). Here, we have identified 15 ESTs relating to scorpion KTxs, among them meuK1, meuK2, meuK3, meuK4, meuK5, meuTx33, and meuTx22 have been firstly described from *M. eupeus* (Additional file: Table S1).

Potassium channel toxins based on their homology, 3D folding pattern, and activity are divided into four families: α-KTx, β-KTx and γ-KTx, κ-KTx (22). From all identified KTXs, meuK1.16, meuK1, meuK2, meuK3, meuK7-3, meuK2-2, meuK5-1, meuK3-1-a, meuK3-1-b, and meuK-toxin were classified in α-KTx, while meuK4 and meuK28-2 were classified in β-KTxs. 

In 1999, leading scientists proposed a so-called unified nomenclature to address and systematize the growing number of known KTx (23). Kalium (http://kaliumdb.org/) is a manually curetted database that all new identified KTXs submitted to UniProt, PDB, NCBI are found in this server (24). So we found the latest submitted peptide in each group of KTXs to see what membership number must be award to our KTXs as their systematic names.

MeuK1.16 is the Eighteenth member of the α-KTx1 family (so its systematic name is α-KTx1.18) containing a domain relating to the Toxin-2 superfamily. This peptide is similar to alpha-KTx 1.16 from Russian *M. eupeus* (C0HJQ8) that is pore blocker of voltage-gated potassium channel rKv1.1/KCNA1 at nanomolar ranges (25). MeuK3 like meuK1.16 has one domain belonging to Toxin-2 superfamily but this toxin is new member of α-KTx15. So, its systematic name is α-KTx15.11.

MeuK3-1-a, meuK3-1-b, and meuK-toxin are new members of α-KTx8; therefore, they are systematically named as α-KTx8.8, α-KTx8.9, and α-KTx8.10, respectively. Both of meuK3-1-a and meuK3-1-b are similar to pMeKTx3-1 from Russian *M. eupeus* (ID: AIL48792) with different similarity (Additional file: Table S1). The pMeKTx3-1 is similar to BmP01 from *Mesobuthus martensii*. The BmP01 potentially inhibits Kv1.3, Kv1.3 and Kv1.1 channels in the mouse and targets the mTRPV1 channel being responsible for pain induction (26). We suggest the meuK3-1-aand the meuK3-1-b as putative pain inducers being good candidates for further researches. Identification of pain-inducing compounds may assist us for eventual pain therapy by scorpion envenomation (26). 

The meuK-toxin has a putative conserved domain belonging to the Toxin-6 superfamily. This family consists of toxin-like peptides isolated from the venom of *Buthus martensii*Karsch scorpion. The peptides containing this domain share close similarity with other scorpion K+ channel toxins. These peptides act by blocking small conductance calcium activated potassium ion channels in their victim (27).

MeuK2-2 is new member of α-KTx9 with systematic name of α-KTx9.13. This toxin like the meuK-toxin shows conserved domain of theToxin-6 superfamily. The meuK2-2 is similar to the pMeKTx2-2 (α-KTx9.2) from Russian *M. eupeus* (ID: AIL48761). The α-KTx9.2 can modify the function of rat KCa2, rat Kv1.1, rat Kv1.2, rat Kv1.3, and Kv1.4 (28).

MeuK1 and the meuK5-1 are new members of α-KTx26, so its systematic name is α-KTx26.4 and α-KTx26.5, respectively. The meuK1 is similar to neurotoxin alpha-KTx 26.2 from *Lychas mucronatus* (ID: D9U2B2) and the meuK5-1 is similar to the pMeKTx5-1 Russian *M. eupeus* (ID: AIL48782).

MeuK2 is similar to the alpha-KTx 14.5 from *Mesobuthus gibbosus* (ID: A0A059U906), and is a new member of the α-KTx14 family with systematic name α-KTx14.6. The alpha-KTx 14.5 is a blocker of Ca^2+^-activated K^+^-channels (KCa1–KCa3) (25).

MeuK7-3 is new member of the KTx17 family with systematic name KTx17.5. This peptide is similar to the pMeKTx7-3 from Russian *M. eupeus* (ID: AIL48764). The pMeKTx7-3 has been reported as homologues of the BmKK4 (25). The BmKK4 is blocker of potassium channels, which inhibits both the delayed rectifier and fast transient potassium current. It is likely that the BmKK4 mainly affects Ca^2+^-activated K^+^ currents (29). According to the similarity, the meuK7-3 could have the same function as potassium channels.

MeuTx22 contains conserved domain of BmKX superfamily. Members of this family assume a structure adopted by the most short-chain scorpion toxins, consisting of a cysteine-stabilized alpha/beta scaffold consisting of a short 3-10-helix and a two-stranded antiparallel beta-sheet. They are predominantly found in short-chain scorpion toxins, and their biological method of action has not, as yet, been defined (27).

The meuK5 and meuTx33 are similar to a putative potassium channel blocker (ID: AAM77698) and neurotoxin BmKX-A1-S31 (ID: Q7Z0H5), respectively, which both are identified in* Mesobuthus martensii* venom. The meuTx33 has no cysteine residue and are categorized in NDBPs.

As mentioned above, meuK4 and meuK28-2 are new members of β-KTxs. meuK4 that is similar to potassium channel toxin Meg-beta-KTx1 from* Mesobuthus gibbosus *(ID: A0A059UI30) (27, 30) classifies in the β-KTx Class 1. This peptide consists of a conserved domain belonging to the Toxin-38 superfamily. This is another family that includes scorpion potassium channel toxins (27). The meuK28-2 is similar to the pMeKTx28-2 from Russian *M. eupeus* (ID: AIL48787) and classifies in the β-KTx Class 2.

*M. eupeus Chloride Channel Toxins*

Three Chlorotoxin-like peptides were identified in the current cDNA library, which were named meuCl14 (ID: KU316183), meuCl15 (ID: KU316184) and meuCl16 (ID: KU316185). Chlorotoxin firstly identified from *Leiurus quinquestriatus *(31). Chlorotoxin and Chlorotoxin-like peptides are known as Chloride channel inhibitor toxins (ClTxs). They are small peptides that block conductance of chloride channels (32). 

MeuCl14 and the meuCl15 are similar to chlorotoxin-2 from *Mesobuthus gibbosus* with different identity), while the meuCl16 is similar to chlorotoxin-3 of *Mesobuthus gibbosus*. Chlorotoxin is a high affinity ligand, which target towards cancer cells, including glioma, melanoma, small cell lung carcinoma, neuroblastoma, and medulloblastoma blocking the small conductance chloride channels (32, 33). Because of much similarity with Chlorotoxin, meuCl14, meuCl15, and meuCl16 may be have the potential in cancer therapy. 

*Other toxins*

Besides the modifiers of ion channels there are some toxins in the scorpion venoms that mostly effect on the central nervous system (CNS). However, few reports are available about them (34). In this study we found nine peptide sequences. They were categorized as “other toxins». meuVNP-1A (ID: KU569304), and meuVNP-1B (ID: KU513853) similar to venom neuropeptide-1 (ID: (ABR21047), meuVNP2 (ID: KU569299) similar to venom neuropeptide-2(ID: (ABR21059) and meuVNP3 (ID: KU569300) similar to venom neuropeptide-3 (ABR21072) effect on neuropeptide signaling pathway, by similarity. meuVNP2 and meuVNP3 by 165 amino acid residues are the longest peptides identified in the Iranian *M. eupeus* venom gland. Both of them contain conserved domain relating to the UPF-0236 superfamily with uncharacterized protein family (27).

meu14toxinA (ID: KU577533) and meu14toxinB (ID: KU569305) are similar to 14 toxin precursor from Russian *M. eupeus* (ID: AJT55733) with 97% and 95% similarity, respectively. Both of them contain conserved domain relating to the Toxin-5 superfamily, containing various secreted scorpion short toxins (27).

meuTx19 (ID: KU569302) similar to the BmKa2 from *Mesobuthus martensii* (ID: Q8N0N8), meuTx20 (ID: KU569306) related to the toxin Tx297 from *Buthus occitanus Israelis* (ACJ23163), meuTx23 (ID: KU513850) comparable to the BmK-YA precursor from *Mesobuthus martensii* (Q9Y0X6), are firstly reported here in the venom of *M. eupeus*. All of them contain a mature peptide of 50 residues, but the length of their signal peptides is different. All of them are categorized as NDBPs.

*Cell Proteins*

Nine transcripts encoding common cellular proteins match proteins involved in diverse cellular processes such as enzymes, cell structure, and other metabolism proteins. They were categorized in “Cell proteins” group. 

Four ESTs (IDs: KU513835-7, KU513842) were identified, which are related to enzymes; Enzymes not only play important role in cell pathways of scorpion, but also some of them contribute to various changes in the pray.

The meuEnz35 (ID: KU513837) is partial CDS of a protein similar to chymotrypsin-like protease-5 from Chinese *M. eupeus* (ID: ABR21066). Chymotrypsin is a Serine protease that cleaves the peptides on the carboxyl side of Phenylalanine, Tyrosine, and Tryptophan residues (35). Moreover, the digestive role of the chymotrypsins has previously been suggested to possess anti-inflammatory and anti-cell-cell adhesion activities. This enzyme has been purified from mammals, fishes, crustaceans and shrimp, but there are few reports in terrestrial arthropods (36). 

meuEnz34 (ID: KU513836) matched significantly with the putative ribonuclease R (RNase R) from *Mesobuthus gibbosus* (AHZ63120). RNase R is a member of the RNR family of exoribonucleases that with 3′-to-5′ hydrolytic exoribonuclease activity plays an important role in degradation of mRNAs and in the quality control of rRNA and Trna (37). MeuEnz34 is partial CDS of RNase R of Iranian *M. eupeus*.

MeuEnz25 and meuEnz26 are partial CDS of two subunits of cytochrome C oxidase (COX), which were found in transcriptome of Iranian *M. eupeus* venom gland. COX is a multi-subunit integral membrane protein located in the inner membrane of mitochondria and the cell membrane of prokaryotes (38). meuEnz25 (ID: KU513835) that contains 91 amino acids residue displayed some similarity with cytochrome C oxidase subunit VIIa from *Rana catesbeiana* (ID: ACO51625). It composed of a conserved domain, Cyt-c-Oxidase-VIIa superfamily. Although, *Rana catesbeiana* is a vertebrate, a study has shown the similarity of vertebrate and invertebrate COX subunit isoforms due to distinct duplication events (39). So, the similarity of meuEnz25 with COXVIIa of a vertebrate is not surprising. 

meuEnz36 (ID: KU513842) is a partial CDS, similar to cytochrome c oxidase subunit III from *Buthus occitanus*. It composed of a conserved domain belong to Heme-copper oxidase _III_like superfamily. Precursors of COX III and COXVIIa have been first reported from *M. eupeus* venom gland.

In addition to enzymes, 5 other ESTs, including meuPep26, meuPep27, meuPep28, meuPep29, and meuPep34 are categorized in “Cell Proteins”. All of themhave been first reported in *M. eupeus* venom gland. meuPep26 (ID: KU513843) is a highly acidic peptide, similar to HAP-1 from *Mesobuthus martensii* (ID: AGV98852). It contains a 28-residues signal peptide linked to a 61-residues mature peptide.

meuPep29 (ID: KU513841) is a NDBP in the “Cell Protein” category. It is partial CDS similar to Paramyosin from *Latrodectus Hesperus* (ADV40180). It contains 152 amino acid residues and consists of a conserved domain family, Myosin_tail_1. This family consists of the coiled-coil myosin heavy chain tail region. The coiled-coil is composed of the tail from two molecules of myosin. These can then assemble into the macromolecular thick filament. The coiled-coil region provides the structural backbone of the thick filament (27). Paramyosin forms a core covered by the myosin in invertebrate muscle thick filaments. The thick filaments of the chelicerate arthropods are similar (but not identical) (40). Similarity of here identified Paramyosin, meuPep29, with Paramyosin of *Latrodectus Hesperus* (77%) corroborates this notion. meuPep29 may be involved in the maintenance of gland structure and/or contractile activity that mediates venom release. 

meuPep27 (ID: KU513844) and meuPep28 (ID: KU513847) are two identified ESTs that are related to metabolism of the lipids. The function of these peptides probably is necessary for the metabolism of lipids in the venom of Iranian *M. eupeus*. MeuPep27 is similar to Lipolysis-activating peptide 1 from* Mesobuthus martensii* (ID: Q6WJF5). From the pharmacological perspective, activation of lipolysis by meuPep27 can be a mechanism for better digestion of lipids in patients with familial apoprotein CII deficiency. Familial apoprotein CII deficiency is a type of primary hyperlipidemia caused by a lack of lipoprotein lipase activator (41). Further investigation will be interesting to unravel whether meuPep27 can be effective in this pathway. 

MeuPep28 is similar to HMG-CoA reductase inhibitor bumarsin from *Mesobuthus martensii* (ID: Q95P90). Statins, currently used for treatment of hyperlipidemia type II, the most common form of hyperlipidemia, are HMG-CoA reductase inhibitors (42). Accordingly, meuPep28, as an HMG-CoA reductase inhibitor is suggested as a candidate for more investigation about using as drug in hyperlipidemia treatment.

*Antimicrobial Peptides*

Four of identified ESTs of the Iranian *M. eupeus* venom are related to Antimicrobial Peptides**. **meuCLAP (ID: AGC92780), meuVAP-6 (ID: E4VP07), and meuAP-18-1 (ID: ADT89761) are similar to other scorpion antimicrobial peptides, while meuVAP-1(ID: KU513848) is similar to venom anionic peptide-1 from Chinese *M. eupeus* (ID: ABR20108). Anionic peptides have been previously reported as highly expressed and conserved peptide among the *Buthidae* scorpion species (43). Although the function of these peptides remains unknown, some researchers have suggested that they might play antimicrobial activity (44) or an important role in pH balance, since neurotoxins are basic peptides (45). 

We had been previously identified the meuCLAP in the venom of Iranian *M. eupeus* venom by Real time RT-PCR (46) however, we found it again in this cDNA library.

Antimicrobial Peptides are small proteins that have wide spectrum activity against pathogens. Selectivity of Antimicrobial Peptides for prokaryotic membranes and their membrane-disruptive mechanisms for which microbes have little natural resistance have caused the attention of researchers in recent years towards the development of novel antibiotics from these peptides (47). Some antimicrobial peptides have been found in scorpion venom. New identified natural antimicrobial peptides are candidate for replacement of the old antibiotics with better performance. Scorpion venom Antimicrobial Peptides are positively charged. Although some of Amphipathic peptides have cystein residue, most of them are NDBPs (6). All Antimicrobial Peptides identified in the current transcriptome contain 47-52 residues (linked to a 22 to 24-amino acids signal peptide) and are categorized as NDBPs.

*Unknown Peptides*

The ORF of three ESTs (meuPep30: KU513838, meuPep31: KU513839, and meuPep34: KU513849) showed similarity by unknown proteins in databases. We classified them in” Unknown Peptides”. 

**Figure 2:**
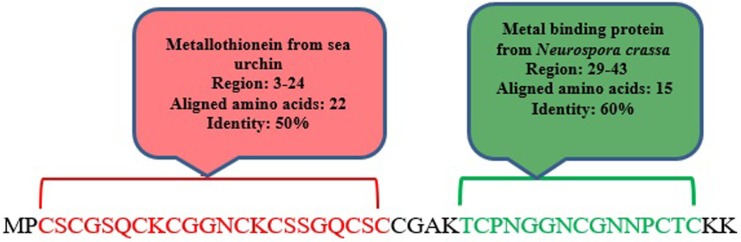
Domain analysis by Phyre 2 server predicted similar domains of meuPep30 with currently known domains

meuPep30, a 45-amino acids peptide with no signal peptide, is similar to hypothetical protein from *Odonthobuthus doriae* (ID: ALX72365). This is a cysteine rich peptide, which has some similarity (91%) with hypothetical secreted protein from *Hottentotta judaicus* (ID: ADY39514). Domain analysis results revealed that meuPep30 has 2 similar domains with known Metallothioneins, as shown in Figure 2. Metallothioneins are intracellular proteins, which are known by their low molecular weight, high cysteine content, lack of aromatic amino acid residues, and binding to 7–12 metal atoms per molecule (48). Studies have indicated this protein is multi-functional, which protect the healthy cells by detoxification of the toxic metals Cd and Hg, homeostatic regulation of essential trace metals Zn and Cu, protection against oxidative stress, neuroprotective mechanisms (49, 50). Increasing of Metallothionein level in embryogenesis of invertebrates may be a tactic for higher survival rate under severs environmental conditions (51). Some investigations were done on mammalian or vertebrate Metallothionein but few studies were directed on invertebrate Metallothionein (52). However, some investigations have focused on Metallothionein amino acid and polynucleotide sequences obtained from terrestrial and aquatic invertebrate (49).

During their long evolutionary existence of scorpion on this planet accompanied by the selective pressure applied on these organisms, they managed to develop series of venom peptides that display diverse biological activities to adapt them to a wide range of environmental conditions (53). So, presence of Metallothionein-like protein in the venom of scorpions seems necessary. The important feature for distinguishing of Metallothioneins is that one-third of their amino acids are cysteine. The length and cysteine content of Metallothionein vary significantly in different organisms (49). MeuPep30 have 45 amino acid length comprising 13 cysteine residues. So about one-third of meuPep30 amino acid components are cysteine. 

Some investigations have reported Metallothionein in some crabs (54). So finding of meuPep30 in Iranian *M. eupeus* is consistent to presence of Metallothionein in *Arthropoda*. It is first time that a Metallothionein-like peptide is reported from a scorpion.

meuPep31 is a 116-amino acids leucine-rich repeat (LRR) peptide. The leucine-rich repeats (LRR)-containing domain is evolutionarily conserved in many proteins associated with innate immunity in plants, invertebrates, and vertebrates(55). These proteins play key roles in neuronal development and maintenance in both invertebrates and vertebrates (56, 57).

**Figure 3:**
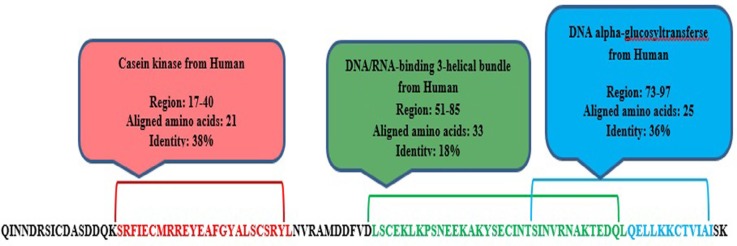
Domain analysis by Phyre 2 server predicted similar domains of meuPep31 with currently known domains

The presence of LRR protein have previously shown in the venom of some *Arthropoda*, like Widow Spiders (58). It was also found in venom gland of the snake, *Agkistrodon blom hoffiisiniticus *(59). Leucine has pivotal role in stability of LRR peptides. Just as domain analysis, meuPep31 have a domain, which in position 17-24 showed 38% similarity with Casein kinase (Figure 3). Some of kinase enzymes, namely leucine-rich repeat kinase, also contain leucine-rich repeat domain. These enzymes similar to Casein kinase isoforms are serine/threonineselective enzymes (60, 61). These results suggest that may be meuPep31 is a leucine-rich repeat enzyme involved in neuronal development or function.

**Figure 4:**
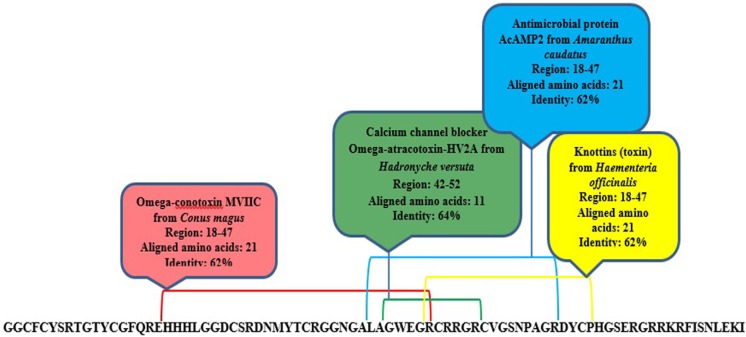
Domain analysis by Phyre 2 server predicted similar domains of meuPep34 with currently known domains

MeuPep34 contains 84 amino acids that form four disulfide bonds in final folding (Additional file: Table S1). It is similar to AbCp-7 from scorpion, *Androctonus bicolor* (87%). The function of AbCp-7 is unknown. Domain analysis of meuPep34 indicated that it contains 4 domains, which are similar to Omega-conotoxinfrom *Conus magus *(62%), Omega-atracotoxin-HV2A from *Hadronycheversuta *(62%), Antimicrobial protein AcAMP2 from *Amaranthuscaudatus* (64%) and Knottins (toxin) (62%) *Haementeriaofficinalis* (Figure 4). The knottin families are fascinating mini-proteins present in many species and featuring various biological actions such as toxic, inhibitory, anti-microbial, insecticidal, cytotoxic, anti-HIV or hormone-like activity. conotoxins and atracotoxins are the most populated knottin families. The protein of this family sharing the inhibitor cystine knots (ICK) scaffold. It was observed in a number of unrelated protein families including, e.g. toxins from plants, bugs, molluscs or arachnids, or antimicrobials from plants, insects or arthropods (62). Omega-conotoxin and Omega-atracotoxin-HV2A are Calcium channel-blocker toxin, while AcAMP2 is an antimicrobial peptide. CaTxs are the key signal transducers of electrical signaling, converting depolarization of the cell membrane to an influx of calcium ions that initiates contraction, secretion, neurotransmission, and other intracellular regulatory events (63). Compared to massive NaTxs and KTxs, only few Calcium channel blocker toxins have been identified from the scorpion venoms (64). A few Calcium channel blocker toxins are also reported from scorpion venom of *Buthidea* family (65, 66). The preliminary results suggest that meuPep34 is a Calcium channel-related toxin with potential antimicrobial activity present in venom gland of Iranian *M. eupeus*.

*Comparative transcriptome analysis of Mesobuthus species*

**Figure 5:**
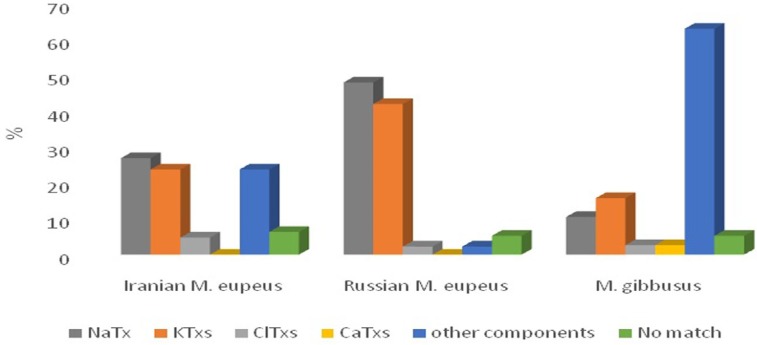
**Relative proportion of the different transcript categories in Iranian** *M. eupeus* and **comparative transcriptome analysis with **those reported from transcriptome analysis by Sanger sequencing in the other sibling species. All transcripts are classified in 5 categories: NaTx (gray bars), KTx (red bars), ClTx (green bars), CaTx (purple bars), other components (comprising other toxins, Antimicrobial Peptides, cell proteins and unknown proteins) (blue bars) and No match sequences (orange bars). **Iranian.*** Mesobuthus eupeus* (this work) *Russian Mesobuthus eupeus *(25). *Mesobuthus gibbosus *(30).

The family *Buthidae*, comprising approximately 500 species, from which transcriptomic analysis of only 13 species have been reported using Sanger sequencing method (**3030**, 43, 45, 67-72). Two studies were related to *Mesobuthus* species including: Russian* M. eupeus* (25) and *M. gibbosus *(30). Transcriptome information of Russian *M. eupeus* was obtained from 133 unique ESTs (from total 244 high-quality ESTs) relating to 64 NaTxs, 56 KTxs, 3ClTxs, 3 other components, and 7 No match sequences, while the transcriptome information of *M. gibbosus* venom component were obtained from 39 unique transcripts (from 177 high-quality ESTs), including 4 NaTxs, 6 KTxs, 1 ClTxs, 1 CaTxs, 24 other components and, 2 No match sequences. We compared percentage of different venom components of Iranian *M. eupeus* with previous reports of transcriptome analysis in two other sibling species of *Mesobuthus* in Figure 5. Comparative transcriptome analysis indicated that in Iranian *M. eupeus* the abundance of NaTx transcripts is high compared to the other toxin categories and congruent with the Russian *M. eupeus species*, while KTxs is the most abundant toxins expressed in the venom of *M. gibussus*. Comparative analysis of *Mesobuthus* species also determined that except for just one CaTx sequence, identified in *M. gibbosus*, any CaTx are found in the venom of *Mesobuthus *species. However, meuPep34, identified in this work, is a candidate for functional assay as putative CaTx. If meuPep34 be a CaTx, it is the first CaTxs reported from *M. eupeus* species.

Despite similar conditions for the library construction, the transcriptome analysis in the species of the*Mesobuthus* genus showed differences in the percentage of venom components categories. However, the gene expression pattern of different components groups related to Iranian *M. eupeus* is nearer to Russian *M. eupeus* compared to *M. gibussus*. 

Comparative analysis of the transcriptome reported for different species of a genus is an important tool for expression analysis of genus specific genes in venom glands, revealing an inter-species difference guiding to different habitat, feeding behavior and other conditions.

## Conclusion

This manuscript is the transcriptome analysis that describes and analyzes different transcripts expressed in the Iranian M. eupeus venom gland, for the first time. We identified several transcripts of toxic and nontoxic. Furthermore, some housekeeping transcripts were obtained. The results of this study in addition to report of the diversity of precursors expressed in Iranian *M. eupeus* venom gland, prepare a large source of information about *M. eupeus* venom components that can provide new insights for further investigations. We identified a putative CaTx peptide (meuPep34) in the venom of *M. eupeus*. However, further infestations must be done for confirming the function of this peptide. Meanwhile, some potential bioactive peptides (such as meuPep27, meuPep28, meuPep30, meuPep31, ClTxs, and Antimicrobial Peptides) described in this work may be an important resource for the investigation and characterization of molecules applicable in pharmaceutical research and biotechnology. Comparison of the venom data of Iranian *M. eupeus* with transcriptomic composition by other *Mesobuthus *species revealed inter-species differences originating from different habitat, feeding, behavior, and other conditions. The results of comparison of transcriptomic composition might be useful for molecular diversity researches.
